# Improved conversion efficiency of amorphous Si solar cells using a mesoporous ZnO pattern

**DOI:** 10.1186/1556-276X-9-486

**Published:** 2014-09-11

**Authors:** Bit-Na Go, Yang Doo Kim, Kyoung suk Oh, Chaehyun Kim, Hak-Jong Choi, Heon Lee

**Affiliations:** 1 Department of Materials Science and Engineering, Korea University, Seoul 136-713, Republic of Korea; 2 Korea Institute of Energy Research, Ulsan 689-798, Republic of Korea

**Keywords:** Amorphous silicon solar cell, Mesoporous ZnO pattern, Nanoimprint lithography, Light scattering

## Abstract

To provide a front transparent electrode for use in highly efficient hydrogenated amorphous silicon (a-Si:H) thin-film solar cells, porous flat layer and micro-patterns of zinc oxide (ZnO) nanoparticle (NP) layers were prepared through ultraviolet nanoimprint lithography (UV-NIL) and deposited on Al-doped ZnO (AZO) layers. Through this, it was found that a porous micro-pattern of ZnO NPs dispersed in resin can optimize the light-trapping pattern, with the efficiency of solar cells based on patterned or flat mesoporous ZnO layers increased by 27% and 12%, respectively.

## Background

An effective light-trapping scheme is an extremely important aspect of Si thin-film solar cell technology, as the inherently thin nature of the light-absorbing layer restricts their long-wavelength light absorption, resulting in a low short-circuit current density (*J*_*sc*_) and power conversion efficiency (PCE) [1-6]. By forming a rough surface morphology on the front transparent electrode, however, the incident light is scattered; thereby increasing the light path length and the probability of light being absorbed within the very thin light-absorbing layer [7-9]. Since the efficiency of this light trapping can be improved by optimizing the front electrode, there is a need to understand the light-trapping capabilities of various surface morphologies. Recently, a variety of light-trapping structures have been created in Si thin-film solar cells through the use of nanoimprint lithography [10,11]. This process is considered one of the most efficient tools for designing light-trapping structures, as it offers a number of advantages in terms of the following: high throughput with a large area, high resolution (~10 nm), simplicity, and low cost [12-16].

In addition to the light-scattering effect of a rough surface, refractive index engineering provides another functional technique for increasing the light absorption within Si thin-film solar cells. This is based on the fact that a large portion of incident light is reflected at the interface formed between layers with a large difference in refractive index, which can be minimized by introducing an additional layer with an intermediate refractive index to create a more gradual change. In this study, we formed mesoporous ZnO pattern on glass substrates for light-scattering effect by using ultraviolet nanoimprint lithography (UV-NIL) [17,18]. The mesoporous ZnO pattern provides strong scattering of light since it has two light-scattering centers. One light-scattering center is optical-function pattern which exhibit excellent light-scattering capabilities. Another light-scattering center is air pores within the mesoporous ZnO layer which significantly enhance light-scattering effect. We fabricated three types of substrates which consisted of flat mesoporous ZnO, mesoporous ZnO pattern, and wet-etched AZO, and performances of a-Si:H thin-film solar cells on three types of substrates were compared to that on flat AZO (reference solar cell).

## Methods

**Figure 1:**
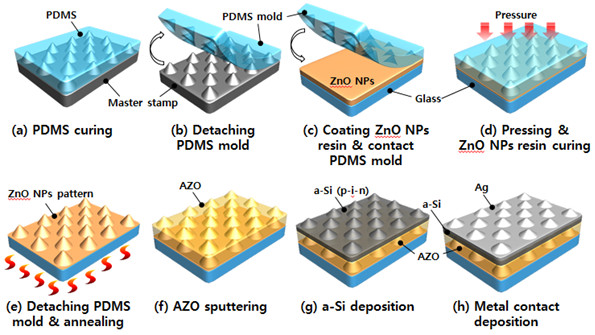
**Schematic showing the various stages in obtaining a mesoporous ZnO pattern.** By UV-NIL using a dispersion of ZnO nanoparticles in resin.

A schematic diagram of the UV-NIL process used is shown in Figure 1. In this, a master stamp was first prepared that consisted of a pattern of micro-cones measuring 2.5 μm in diameter and 1.5 μm in height [19-21]. A replica mold of this master stamp was then fabricated by UV nanoimprinting of polydimethylsiloxane (PDMS), which was formulated using a 10:1 volumetric ratio mixture of Sylgard 184A (PDMS base, Dow Corning Co., Midland, MI, USA) and Sylgard 184B (PDMS curing agent, Dow Corning Co., Midland, MI, USA). Sylgard 184A and Sylgard 184B were purchased from Dow Corning Co. This mixture was poured onto the master mold and degassed for 20 min prior to curing on a hot plate at 120°C for 2 h (Figure 1a,b) [22-24].

In order to increase the adhesion between the glass substrate and ZnO NP resin, the glass surface was subjected to UV-ozone treatment for 5 min to remove impurities and render it hydrophilic [25]. The ZnO NP resin dispersion was formulated using a mixture of 10 g of benzyl methacrylate (BzMA) (Sigma-Aldrich, St. Louis, MO, USA) monomer, 8 g of ZnO NP solution (Ditto Technology, Gyeonggi-do, Seoul, South Korea, <130 nm, ethanol base 40 wt%), and 2 g of UV photoinitiator (Irgacure 184) (Sigma-Aldrich, St. Louis, MO, USA); which was then spin-coated onto the glass substrate at rate of 2,000 rpm for 30 s (Figure 1c).

The PDMS replica mold was immediately contacted with the spin-coated ZnO NP resin and then held at pressure of 5 bar for 15 min to ensure vaporization of the resin solvent complete filling of the PDMS replica mold. Next, the PDMS replica mold was exposed to UV radiation for 20 min to indurate the ZnO NP resin pattern and was then removed (Figure 1d) [26]. By UV nanoimprinting the PDMS mold into the ZnO NP layer, a high-fidelity replication of the master stamp pattern was achieved. The ZnO NP pattern was then annealed at 500°C for 1 h to remove any remaining solvent and impurities (Figure 1e) [27], leaving a mesoporous ZnO pattern on glass. Finally, an AZO layer was deposited onto this mesoporous ZnO pattern by RF magnetron sputtering, using an AZO target composed of 98% ZnO and 2% Al_2_O_3_ (Figure 1f).

To study the effects of the mesoporous ZnO pattern on the performance of a-Si:H thin-film solar cells, 250-nm-thick single-junction a-Si p-i-n layers were deposited onto the prepared electrode by plasma-enhanced chemical vapor deposition (PECVD) (Figure 1g). The p-type layer was deposited using SiH_4_, H_2_, and B_2_H_6_; the intrinsic layer (i-layer) was deposited using a mixture of H_2_ and SiH_4_ gases; and the n-type layer was deposited using SiH_4_, H_2_, and PH_3_. To complete the cell, an Ag electrode was deposited onto the a-Si p-i-n layers by a thermal evaporator (Figure 1h).

The surface morphology of the patterned ZnO NP layers was characterized by scanning electron microscopy (SEM) (Horiba EX-200, Horiba, Minami-Ku, Kyoto, Japan) and atomic force microscopy (AFM) (XE-100). Optical characterization of the four substrates (flat AZO, flat AZO with flat mesoporous ZnO, AZO with mesoporous ZnO pattern, and wet-etched AZO) was performed using a UV-visible spectrometer equipped with an integrating sphere (Jasco V-650, Easton, MD, USA). Cross-sectional images of the a-Si:H thin-film solar cells were obtained by focused-ion-beam SEM (FIB-SEM, FEI Nova 600 Dual Beam FIB, Center for Electron Microscopy and Analysis, Columbus, OH, USA). Photocurrent density-voltage (J–V) measurements of the cells were performed using a solar simulator under standard test conditions (25°C, AM 1.5, 100 mW/cm^2^) and from the external quantum efficiency (EQE) measured at zero bias.

## Results and discussion

**Figure 2:**
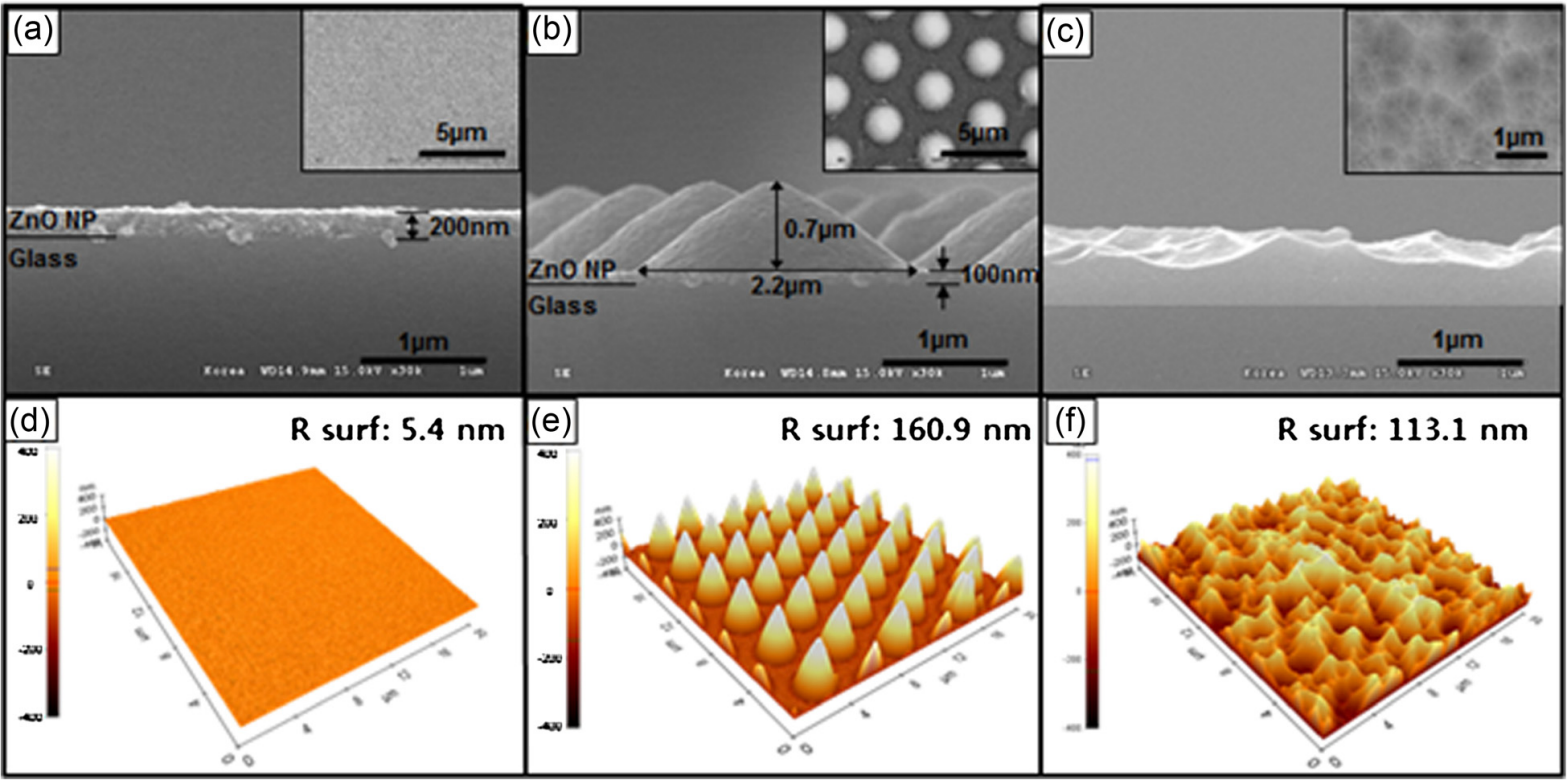
**SEM images of the layers.** SEM images of **(a)** flat mesoporous ZnO layer, **(b)** mesoporous ZnO pattern, and **(c)** wet-etched AZO (insets: top views). **(d-f)** AFM images of a 20 × 20 μm area of the substrates.

**Figure 3:**
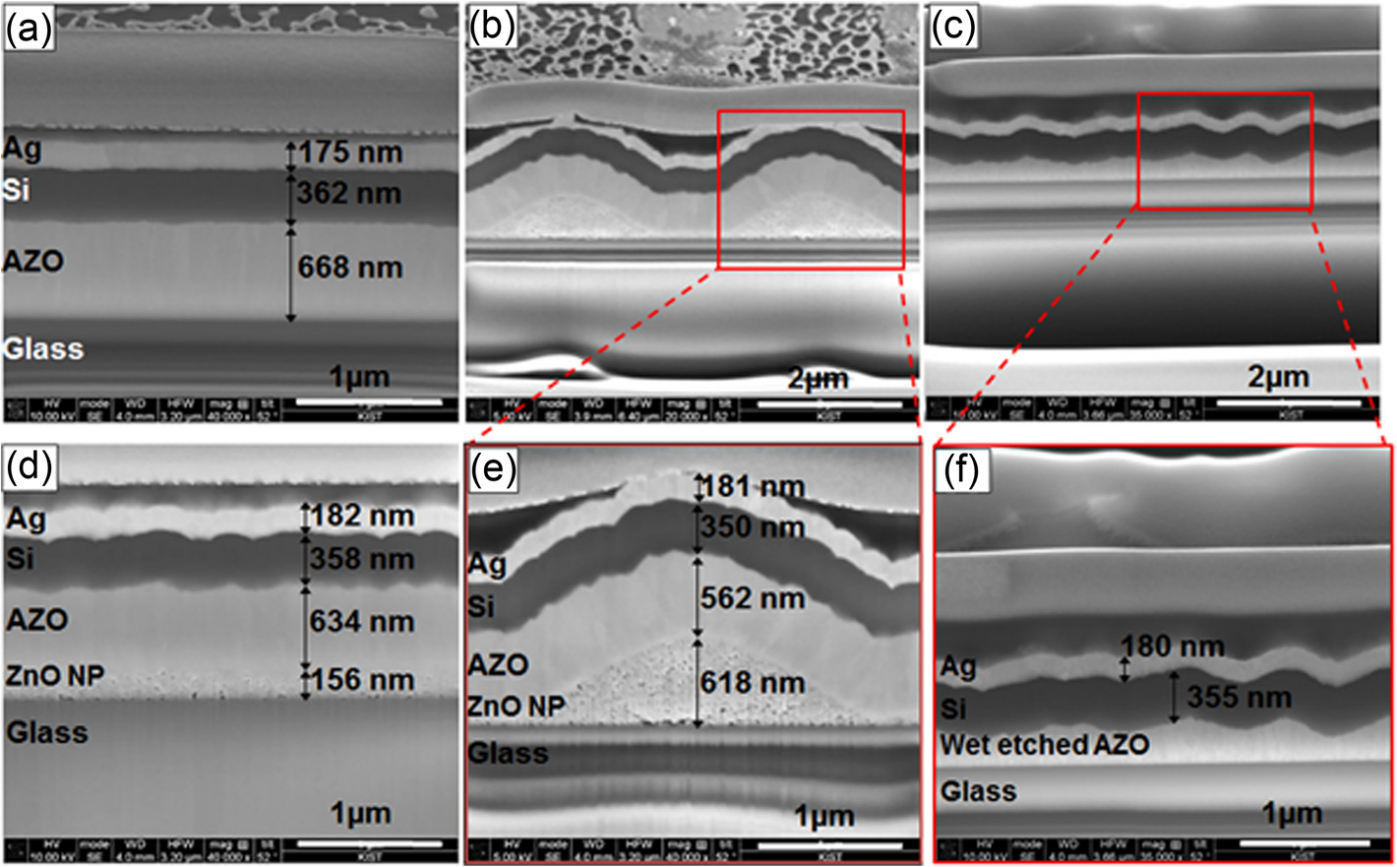
**FIB-SEM cross-sectional images of a-Si:H thin-film solar cells grown on the following. (a)** flat AZO, **(d)** flat mesoporous ZnO layer, **(band e)** patterned mesoporous ZnO, or **(cand f)** wet-etched AZO substrates.

**Figure 4:**
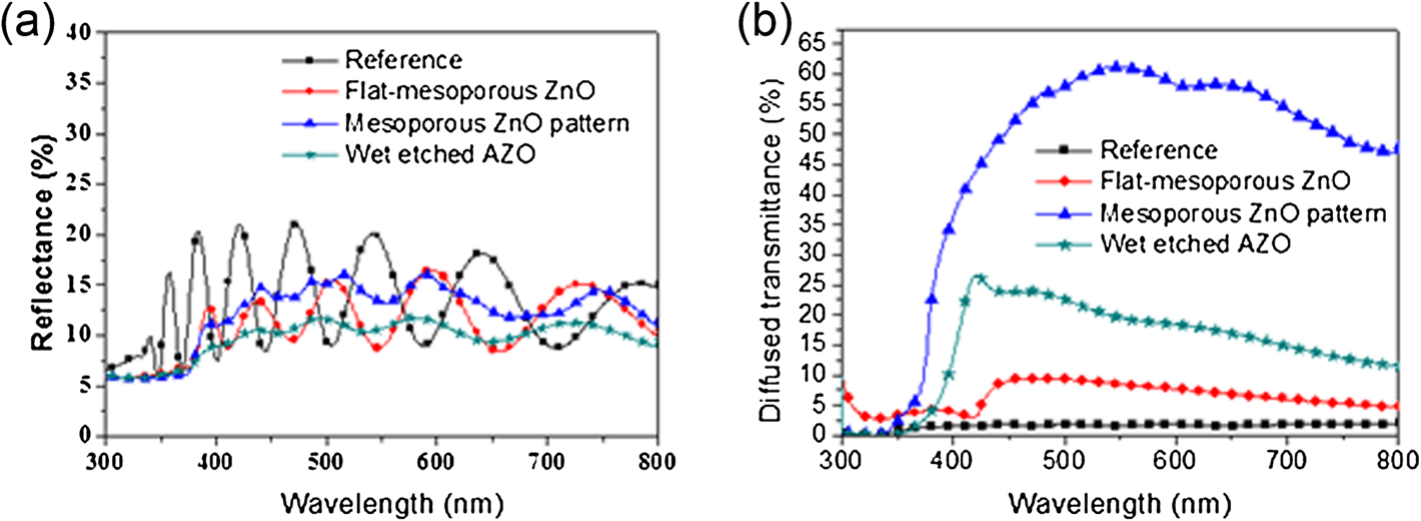
**Reflectance and diffused transmittance of substrates. (a)** Reflectance and **(b)** diffused transmittance of different substrates after AZO deposition.

Figure 2a,d shows, respectively, the SEM and AFM images obtained of the flat mesoporous ZnO layer produced on glass by spin coating and annealing. In Figure 2b,e, we see that that the subsequent UV-NIL process successfully replicates the pattern of the master stamp with a high degree of fidelity; however, the pattern size is shrunk due to the solvent in the ZnO NP dispersion resin being absorbed into the PDMS mold and removed during the UV-NIL process [17]. Thus, in the mesoporous ZnO pattern, the diameter and height of the micro-cone-shaped structure are reduced to 2.2 and 0.7 μm, respectively. Finally, Figure 2c,f shows the randomly patterned AZO produced by wet etching with dilute HCl.Figure 3a,d shows that interfaces in an amorphous silicon solar cell based on flat AZO glass and a flat-mesoporous ZnO glass are similarly flat; whereas those of cells based on mesoporous ZnO pattern and wet-etched AZO are distinctly rough (Figure 3b,c). These roughened surfaces cause a scattering of light at the interface that extends the optical path, with the gas pores in the mesoporous ZnO also functioning as a secondary light-scattering center.From the optical properties of the four AZO substrate types shown in Figure 4, it is clear that their reflectance values are almost comparable to, or slightly less than, the reference sample. The slightly reduced reflectance of the mesoporous ZnO layers is attributed to the presence of gas pores, which cause a gradual change from 1.5 to 1.7, 1.9, and 4.5 (at 550 nm) with a shift from glass to mesoporous ZnO, AZO, and amorphous silicon, respectively. Since this fluctuation in reflectance originates from the AZO layer, it changes in response to the surface roughness of this layer. Furthermore, the diffused transmittance of the mesoporous ZnO layers is increased due to the light scattering of the gas pores, as seen in Figure 4b. Thus, the high diffused transmittance of the patterned mesoporous ZnO of up to 63% is caused by a combination of light scattering by the pattern morphology and the pores. With the wet-etched AZO, on the other hand, the diffused transmittance is increased up to 25% in the 430 wavelength range due to the light scattering of its random pattern. This means that the light-scattering capability of mesoporous ZnO pattern is superior within a wavelength region of 370 to 800 nm.

**Figure 5:**
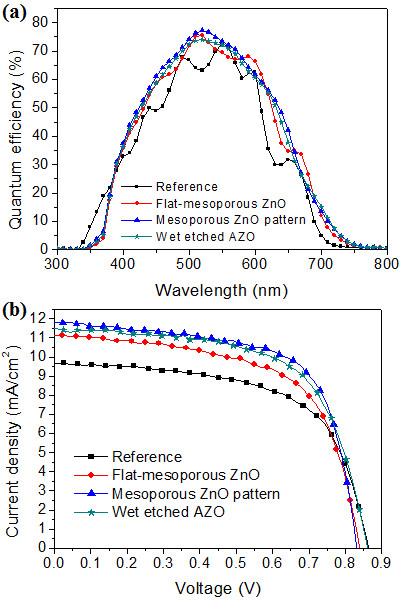
**Characteristics of a-Si:H thin-film solar cells. (a)** External quantum efficiency and **(b)** current-voltage characteristics of a-Si:H thin-film solar cells prepared on different electrodes.

Figure 5 shows the EQE and J-V characteristics for the various a-Si:H thin-film solar cells prepared in this study, with their respective J-V parameters summarized in Table 1. As expected from the optical properties of the four electrodes, the *J*_*sc*_ values of the a-Si:H thin-film solar cells prepared on mesoporous ZnO or wet-etched AZO are higher than the reference cell due to either the effect of inserting a pattern layer or the presence of pores. In the case of the mesoporous patterned ZnO pattern, however, the strong light scattering generated by the combination of a micro-cone patterned surface and pores results in a higher *J*_*sc*_ than the other substrates, which have only a single light-scattering center.

**Table 1:** Characteristics of a-Si:H thin-film solar cells grown on four different substrates

**Substrate**	**V**_**oc**_**[V]**	**J**_**sc**_**[mA/cm**^**2**^**]**	**∆J**_**sc**_**[%]**	**FF [%]**	**Efficiency [%]**	**∆efficiency [%]**
Reference	0.86	9.68	Standard	61.14	5.11	Standard
Flat mesoporous ZnO	0.84	11.13	+15.0	61.19	5.72	+11.94
Mesoporous ZnO pattern	0.83	11.82	+22.1	65.83	6.47	+26.61
Wet-etched AZO	0.86	11.55	+19.3%	60.81	5.99	+17.22

From Figure 5b and Table 1, it is evident that the fill factor (FF) of a-Si:H thin-film solar cells on mesoporous patterned ZnO and wet-etched AZO is increased, despite the rougher surface of the AZO layer. This can be attributed to the short charge transport path within the cells, in that the thickness of the layer in the direction of the local surface normal is less than in the deposition direction. This shorter charge transport path lowers the series resistance, thus increasing the FF.

## Conclusions

Two different types of light-scattering centers were successfully produced in the form of an optical-function pattern and air pores in mesoporous ZnO and were shown to increase the light path in the absorption layer and the light absorption capability. The combination of both light-scattering centers in patterned mesoporous ZnO produced better performance the single center present in flat mesoporous ZnO or wet-etched AZO substrates. Nevertheless, a-Si:H thin-film solar-cells based on any one of these three types of substrate exhibit a higher *J*_*sc*_ and conversion efficiency than a cell based on a flat substrate; though the best performance is achieved with a mesoporous ZnO pattern, with a 22.1% and 26.6% increase in *J*_*sc*_ and conversion efficiency, respectively.

## Competing interests

The authors declare that they have no competing interests.

## Authors' contributions

BNG and YDK carried out the experiments and finalized the manuscript. KSO, CHK, and HJCYK participated in the design of the study and helped draft the manuscript. HL supervised the work. All authors read and approved the final manuscript.
